# Supplementary material for: Characterization of a fold in TANGO1 evolved from SH3 domains for the export of bulky cargos
Source: Nat Commun. 2023 Apr 20;14:2273. doi: 10.1038/s41467-023-37705-4 (PMC10119292; doi:10.1038/s41467-023-37705-4)
Supplement: Supplementary file 1 — Supplementary Information [file 41467_2023_37705_MOESM1_ESM.pdf]

SUPPLEMENTAL INFORMATION

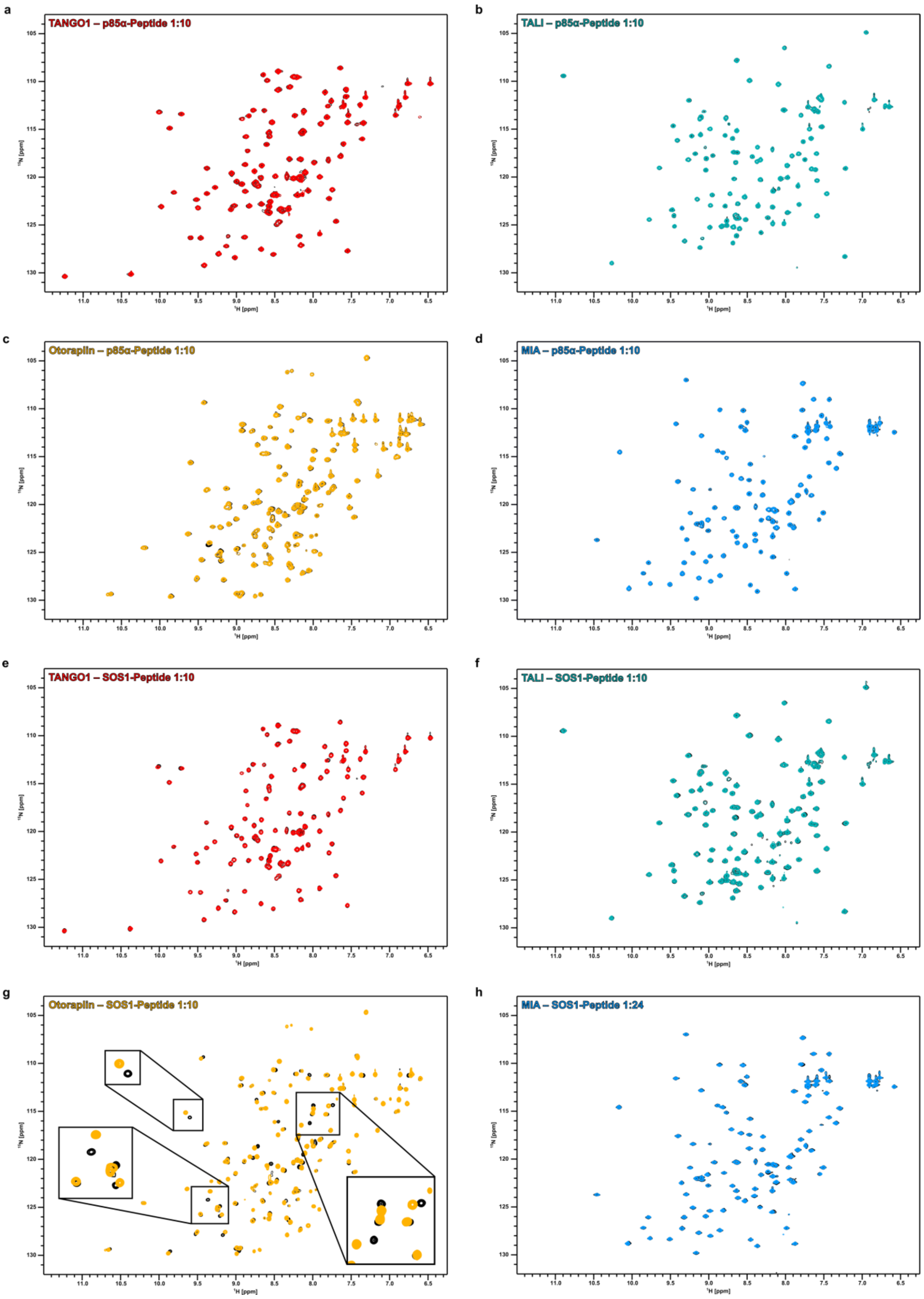

Supplementary Figure 1

$^{1}H^{15}N$-HSQC titration spectra of class I and II PPII ligands with homologous cargo-recognition domains encoded by the *mia* gene family.

a-d, Titration spectra of MOTH domains from TANGO1 (red), TALI (cyan), Otoraplin (yellow), and MIA (blue), respectively, with an exemplary class I PPII ligand. The peptide was derived from residues 91 to 104 of the phosphatidylinositol-3-kinase regulatory subunit alpha (p85α) known to interact with the SH3 domain of human tyrosine-kinase Fyn. [1] Reference spectra for all domains are shown in black. **e-h**, Analogous titration spectra with a class II PPII ligand. The peptide sequence corresponds to residues 1149 to 1158 of the guanidine exchange factor Son-of-Sevenless 1 (SOS1) of the Ras protein, reported to bind to the N-terminal SH3 domain of the growth factor receptor-bound protein 2 (Grb2). [2] A selection of significant CSPs for the Otoraplin/SOS1 titration is highlighted in **g**.

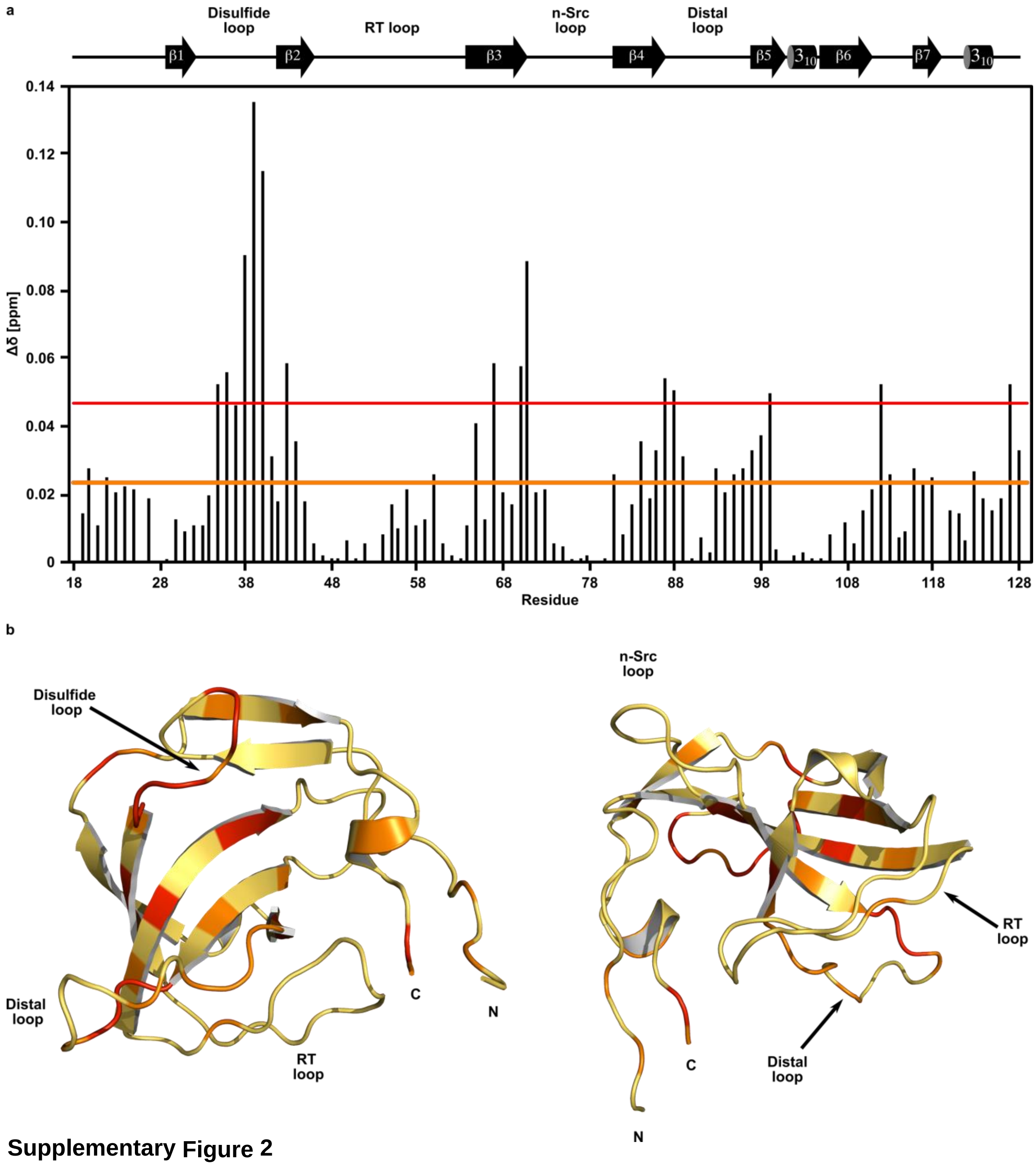

Supplementary Figure 2

CSP analysis of Otoraplin's interaction with a class II PPII ligand.

a, Chemical shift differences between a reference spectrum and a tenfold molar excess of peptide plotted against the amino acid sequence. Source data are provided as a Source Data file. Orange and red line indicate the single and double standard deviation (SD), respectively, based on the average shift difference for all residues. **b**, Chemical shift differences exceeding the single (orange) or double (red) SD projected onto predicted structure of Otoraplin by AlphaFold (AF-Q9NRC9-F1

[https://alphafold.ebi.ac.uk/entry/Q9NRC9]. Accessed via https://alphafold.ebi.ac.uk/). Otoraplin exhibited a very weak interaction with class II ligands, and CSP mapping revealed the interaction site to be located opposite to the canonical SH3 binding groove between the RT and nSrc loop**.** Moreover, the interaction appears to be mainly mediated by electrostatic interactions between arginine side chains of the peptide and negative charges of the disulfide and distal loop of Otoraplin.

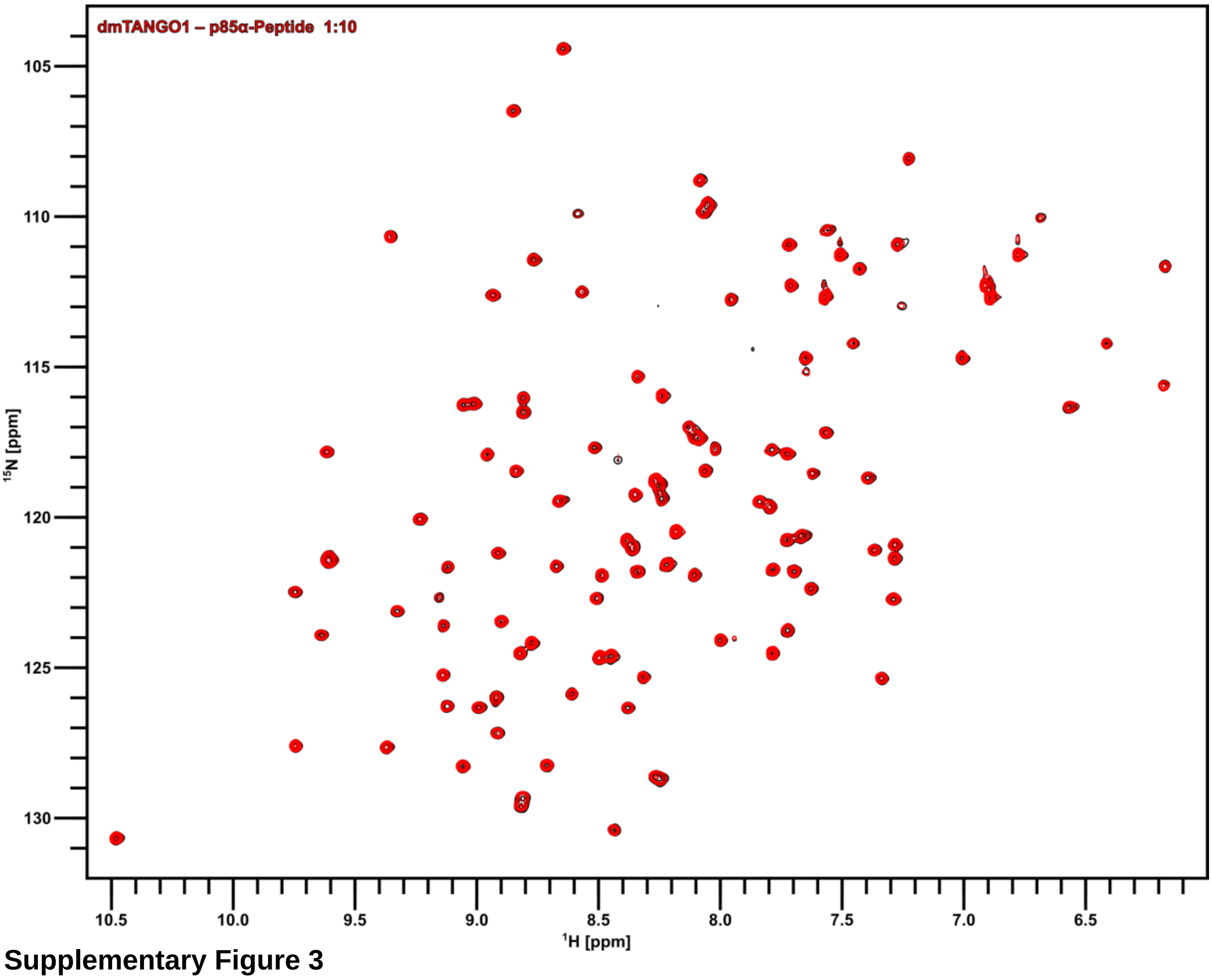

Supplementary Figure 3

^{1}H^{15}N-HSQC of dmTANGO1(30-139) and titration spectrum of PPII class I peptide.

Titration spectra of TANGO1's cargo-recognition domain from *D. melanogaster* with an exemplary class I PPII ligand. The peptide was derived from residues 91 to 104 of the phosphatidylinositol-3-kinase regulatory subunit alpha (p85α) known to interact with the SH3 domain of human tyrosine-kinase Fyn. [1] Reference spectrum is shown in black.

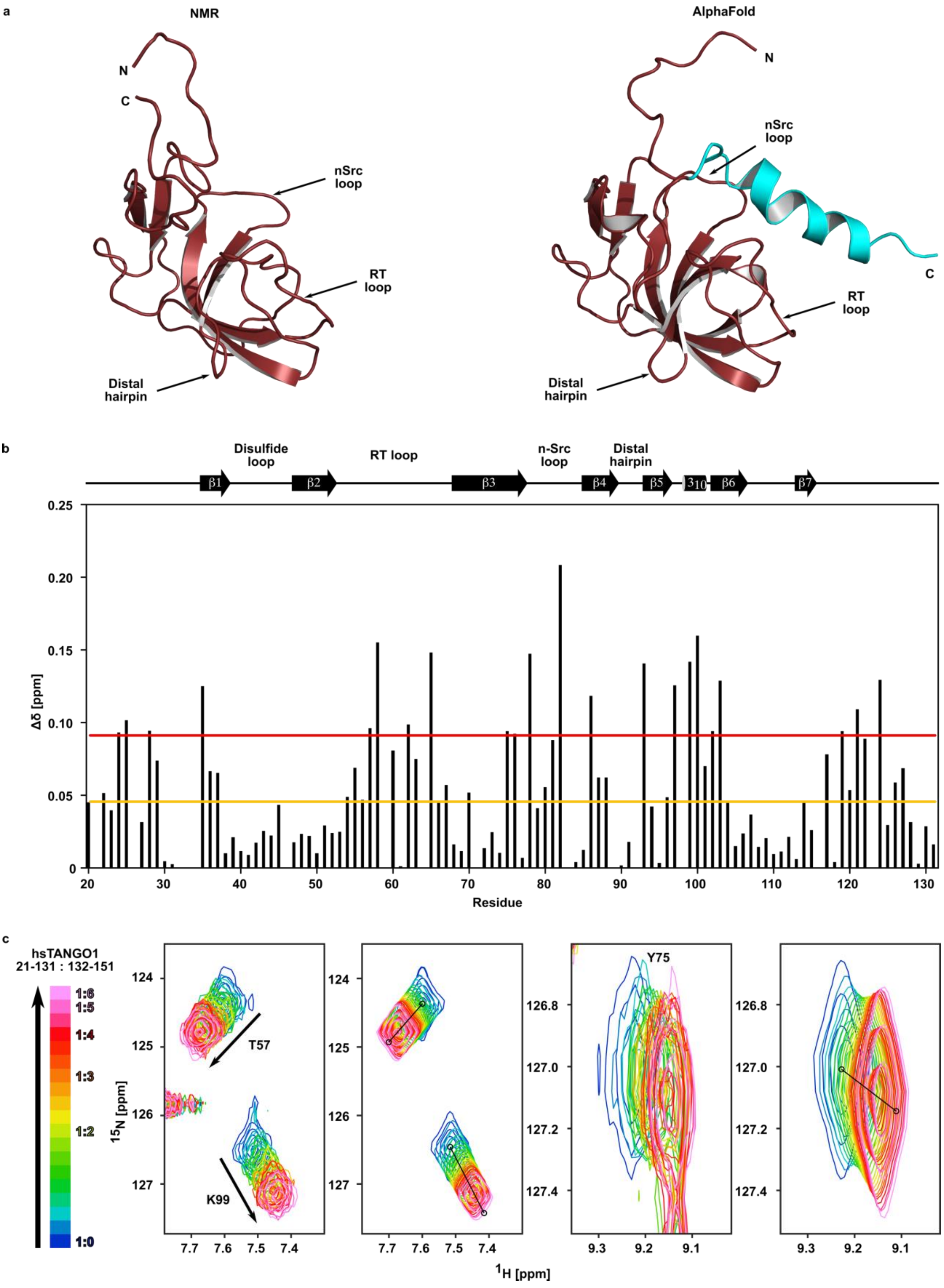

a
NMR
N
C
nSrc loop
RT loop
Distal hairpin
AlphaFold
N
nSrc loop
C
RT loop
Distal hairpin
b
Disulfide loop
RT loop
n-Src loop
Distal hairpin
β1
β2
β3
β4
β5
3₁₀
β6
β7
Δδ [ppm]
Residue
c
hsTANGO1 21-131 : 132-151
1:6
1:5
1:4
1:3
1:2
1:0
T57
K99
Y75
15N [ppm]
1H [ppm]

Supplementary Figure 4

Comparison of TANGO1's MOTH domain's structure from NMR spectroscopy and AlphaFold's structure prediction.

a, Structures of TANGO1's MOTH domain from structure determination by solution NMR spectroscopy and prediction by AlphaFold (AF-Q5JRA6-F1 [https://alphafold.ebi.ac.uk/entry/Q5JRA6]) display an RMSD for the structured region excluding the C-terminal helix of the prediction (cyan) of 0.84 Å. Most differences can be observed for non-secondary structure elements, predominantly for the nSrc loop and region between β-strands six and seven. These exhibit dynamic movements on the pico- to nanosecond timescale according to the hetNOE (Figure 2). **b**, Chemical shift differences observed for the human TANGO1 (21-131) upon titration of a peptide corresponding to residues 132-151 (displayed in **a** in cyan) with a sixfold molar excess. Source data are provided as a Source Data file. Single and double SD based on the average shift difference for all residues are indicated by orange and red line, respectively. **c** Exemplary excerpts of two-dimensional lineshape analysis of the interaction between human TANGO1(21-131) and a peptide corresponding to residues 132 to 151 of human TANGO1 using TITAN. Real spectra are displayed in respective left panels, re-calculated spectra based on fitted parameters are shown in the right panels.

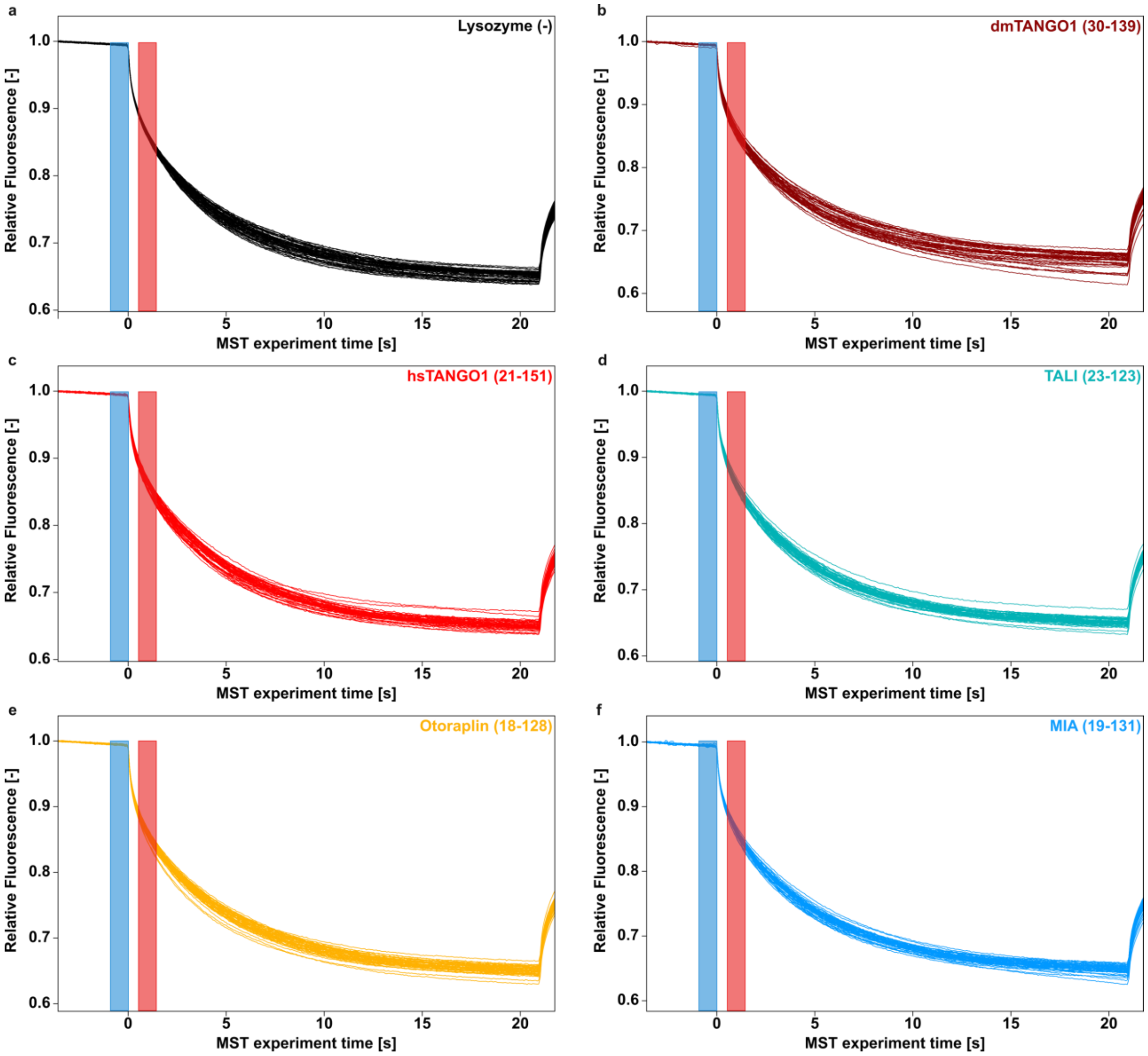

Supplementary Figure 5

MST traces of various *mia* gene members with labeled type IV collagen

Lysozyme served as a negative control. MST-off times used for analysis are indicated with blue, on times by red rectangles**.** Traces of capillaries displaying aggregation of adsorption were excluded for final analysis and are not shown. Source data are provided as a Source Data file.

Supplementary Table 1.

Statistics for all conformational restraints used in the calculation and geometric quality statistics for the final NMR ensemble of the 20 lowest energy structures for TANGO1's cargo-recognition domain. Deviations ± standard deviations of this ensemble from averaged coordinates are summarized in the RMSD.

**Conformational restraints**	
Distance restraints	
Intraresidual (i = j)	815
Sequential (|i - j| = 1)	314
Short-range (2 < |i - j| < 3)	69
Medium-range (4 < |i - j| < 5)	27
Long-range (|i - j| ≥ 5)	404
Ambiguous	175
Dihedral restraints (Φ/Ψ)	195
Other structural restraints	
Oxidized cysteines	4
cis-prolines	1
**Total number of restraints**	**2004**

**Structure quality**	
Average RMSD of secondary structures	[Å]
Backbone	0.29 ± 0.05
Heavy atoms	0.64 ± 0.08
Ramachandran statistics	[%residues]
Core regions	80.1
Allowed regions	18.3
Generous regions	0.7
Disallowed regions	1.0

Supplementary Table 2.

Parameters extracted from microscale thermophoresis dilution series

Protein	K_D [μM]	K_D confidence [μM]	n	Signal to noise
Lysozyme	n.d.	n.d.	4	n.d.
dmTANGO1(30-139)	6.9	3.2	3	7.54
hsTANGO1(21-151)	3.3	1.2	3	10.4
TALI(23-123)	11.4	4.8	3	11.8
Otoraplin(18-128)	9.9	2.9	3	17.1
MIA(19-131)	n.d.	n.d.	3	n.d.

Supplementary Table 3.

Pulse programs used for NMR spectroscopy.

Pulse programs and parameters such as number of scans (NS), amount of recorded data points using non-uniform sampling (NUS), sweep width (SW), and points recorded in the time domain (TD) used for NMR spectroscopy.

Pulse program			F1		F2		F3		F4		Ref
	NS	NUS	SW [ppm]	TD	SW [ppm]	TD	SW [ppm]	TD	SW [ppm]	TD	
zggpw5	8	-	15.9	32768	-	-	-	-	-	-	[3]
hsqcfpf3gpphwg	8	-	29.4	256	15.9	2048	-	-	-	-	[4–7]
hsqcctetgpsp	32	-	80.0	256	13.0	1024	-	-	-	-	[8]
hncagpwg3d	16	-	32.0	128	29.4	48	15.9	2048	-	-	[9–11]
hncacbgpwg3d	128	30%	80	128	29.4	40	15.9	2048	-	-	[12,13]
hncogpwg3d	16	-	9.0	128	29.4	40	15.9	2048	-	-	[9–11]
hncacogpwg3d	64	30%	9.0	128	29.4	40	15.9	2048	-	-	[11,14]
cbcaconhgp3d	64	30%	80.0	128	29.4	40	15.9	2048	-	-	[13,15]
hncocacbgpwg3d	16	25%	80.0	128	30.0	40	15.9	2048	-	-	[16]
hccconhgpwg3d3	64	25%	80.0	128	35.0	40	15.9	2048	-	-	[17–22]
noesyhsqcf3gpwg3d	32	-	14.0	128	29.4	40	15.9	2048	-	-	[23]
hnhagp3d	32	-	29.4	40	14.0	128	15.9	2048	-	-	[24,25]
hccconhgpwg3d2	64	25%	15.9	128	35.0	40	15.9	2048	-	-	[17–22]
noesyhsqcetgp3d	32	-	14.0	128	80.0	64	14.0	2048	-	-	[26]
hcchcogp3d	16	-	14.0	128	80.0	64	14.0	2048	-	-	[27]
hcchdigp3d	16	-	14.0	128	80.0	64	14.0	2048	-	-	[27]
hsqcnoesyhsqccngp4d	8	30%	80.0	32	10.0	64	35.0	32	15.9	2048	[28]
hsqcnoesyhsqcccgp4d	8	-	80.0	32	10.0	64	80.0	32	15.9	2048	[13,23,29,30]
hsqcnoef3gpsi3d	32	-	35.0	2	29.4	256	15.9	2048	-	-	[23,31]

Supplementary Table 4.

Parameters used for calculations with ARIA 2.3.1 for the structure determination of TANGO1's cargo-recognition domain.

**General settings**	
Assignment frequency window	[ppm]
^{1}H	0.04
$^{13}C/^{15}N$	0.5
**Step 1 – Assignment and unrefined structure calculation**	
Violation tolerance [Å]	
Iteration 0	5.0
Iteration 1	4.0
Iteration 2	3.0
Iteration 3 – 5	1.0
Iteration 6 – 8	0.5
Number of conformers	calculated/analyzed
Iteration 0 – 4	800/20
Iteration 5 – 6	200/20
Iteration 7 – 8	200/7
Structures with no violations in iteration 8	33
Simulated-annealing	
Distance restraint potential	log-harmonic
#steps high-temperature	20,000
#steps cooling 1	10,000
#steps cooling 2	8,000
**Step 2 – Structure refinement**	
Violation tolerance [Å]	
Iteration 0	0.5
Number of conformers	calculated/analyzed
Iteration 0	6,000/50
Refined structures	200
Structures with no violations after refinement	76
Simulated-annealing	
Distance restraint potential	flat-bottom
#steps high-temperature	10,000
#steps cooling 1	20,000
#steps cooling 2	16,000

Supplementary References

1. Morton, C. J. *et al.* Solution structure and peptide binding of the SH3 domain from human Fyn. *Structure* **4**, 705–714 (1996).
2. Wittekind, M. *et al.* Solution structure of the Grb2 N-terminal SH3 domain complexed with a ten-residue peptide derived from SOS: Direct refinement against NOEs, J-couplings and 1H and 13C chemical shifts. *J. Mol. Biol.* **267**, 933–952 (1997).
3. Liu, M. *et al.* Improved Watergate Pulse Sequences for Solvent Suppression in NMR Spectroscopy. *J. Magn. Reson.* **132**, 125–129 (1998).
4. Bodenhausen, G. & Ruben, D. J. Natural abundance nitrogen-15 NMR by enhanced heteronuclear spectroscopy. *Chem. Phys. Lett.* **69**, 185–189 (1980).
5. Piotto, M., Saudek, V. & Sklen, V. Gradient-tailored excitation for single-quantum NMR spectroscopy of aqueous solutions. **2**, 661–665 (1992).
6. Sklenár, V., Piotto, M., Leppik, R. & Saudek, V. Gradient-Tailored Water Suppression for 1H-15N HSQC Experiments Optimized to Retain Full Sensitivity. *J. Magn. Reson.* **102**, 241–245 (1993).
7. Mori, S., Abeygunawardana, C., Johnson, M. O. & van Zijl, P. C. M. Improved Sensitivity of HSQC Spectra of Exchanging Protons at Short Interscan Delays Using a New Fast HSQC (FHSQC) Detection Scheme That Avoids Water Saturation. *J. Magn. Reson.* **108**, 94–98 (1995).
8. Vuister, G. W. & Bax, A. Resolution Enhancement and Spectral Editing of Uniformly 13C-Enriched Proteins by Homonuclear Broadband 13C Decoupling. *J. Magn. Reson.* **98**, 428–435 (1992).
9. Grzesiek, S. & Bax, A. Improved 3D triple-resonance NMR techniques applied to a 31 kDa protein. *J. Magn. Reson.* **96**, 432–440 (1992).
10. Schleucher, J., Sattler, M. & Griesinger, C. Coherence Selection by Gradients without Signal Attenuation: Application to the Three-Dimensional HNCO Experiment. *Angew. Chemie Int. Ed. English* **32**, 1489–1491 (1993).
11. Kay, L. E., Xu, G. Y. & Yamazaki, T. Enhanced-Sensitivity Triple-Resonance Spectroscopy with Minimal H2O Saturation. *Journal of Magnetic Resonance, Series A* vol. 109 129–133 (1994).
12. Wittekind, M. & Mueller, L. HNCACB, a High-Sensitivity 3D NMR Experiment to Correlate Amide-Proton and Nitrogen Resonances with the Alpha- and Beta-Carbon Resonances in Proteins. *Journal of Magnetic Resonance, Series B* vol. 101 201–205 (1993).

13. Muhandiram, D. R. & Kay, L. E. Gradient-Enhanced Triple-Resonance Three-Dimensional NMR Experiments with Improved Sensitivity. *Journal of Magnetic Resonance, Series B* vol. 103 203–216 (1994).

14. Clubb, R. T., Thanabal, V. & Wagner, G. A Constant-Time Three-Dimensional Tripel-Resonance Pulse Scheme to Correlate Intraresidue 1HN, 15N, and 13C' Chemical Shifts in 15N-13C-Labeled Proteins. *J. Magn. Reson.* **97**, 213–217 (1992).

15. Grzesiek, S. & Bax, A. Amino acid type determination in the sequential assignment procedure of uniformly 13C / 15N-enriched proteins. **3**, 185–204 (1993).

16. Yamazaki, T., Muhandiranv, D. R., Kay, L. E., Lee, W. & Arrowsmith, C. H. A Suite of Triple Resonance NMR Experiments for the Backbone Assignment of 15N, 13C, 2H Labeled Proteins with High Sensitivity. *J. Am. Chem. Soc.* **116**, 11655–11666 (1994).

17. Montelione, G. T. *et al.* An Efficient Triple Resonance Experiment Using Carbon-13 Isotropic Mixing for Determining Sequence-Specific Resonance Assignments of Isotopically Enriched Proteins. *J. Am. Chem. Soc.* **114**, 10974–10975 (1992).

18. Grzesiek, S., Anglister, J. & Bax, A. Correlation of backbone amide and aliphatic side-chain resonances in 13C/15N-enriched proteins by isotropic mixing of 13C magnetization. *J. Magn. Reson. Ser. B* (1993).

19. Logan, T. M., Olejniczak, E. T., Xu, R. X. & Fesik, S. W. A general method for assigning NMR spectra of denatured proteins using 3D HC(CO)NH-TOCSY triple resonance experiments. *J. Biomol. NMR* **3**, 225–231 (1993).

20. Clowes, R. T., Boucher, W., Hardman, C. H., Domaille, P. J. & Laue, E. D. A 4D HCC(CO)NNH experiment for the correlation of aliphatic side-chain and backbone resonances in 13C/15N-labelled proteins. *J. Biomol. NMR* **3**, 349–354 (1993).

21. Carlomagno, T. *et al.* PLUSH TACSY: Homonuclear planar TACSY with two-band selective shaped pulses applied to Cα,C′ transfer and Cβ,Caromatic correlations. *J. Biomol. NMR* **8**, 161–170 (1996).

22. Lyons, B. A. & Montelione, G. T. An HCCNH Triple-Resonance Experiment Using Carbon-13 Isotropic Mixing for Correlating Backbone Amide and Side-Chain Aliphatic Resonances in Isotopically Enriched Proteins. *Journal of Magnetic Resonance, Series B* vol. 101 206–209 (1993).

23. Parella, T. Pulse Program Catalogue:II. Biomolecular NMR Experiments. *Pulse Progr. Cat.* 1–

249 (2006).

24. Vuister, G. W. & Bax, A. Quantitative J Correlation: A New Approach for Measuring Homonuclear Three-Bond J(HNHα) Coupling Constants in 15N-Enriched Proteins. *J. Am. Chem. Soc.* **115**, 7772–7777 (1993).

25. Vuister, G. W. & Bax, A. Measurement of four-bond HN-Hα J-couplings in staphylococcal nuclease. *J. Biomol. NMR* **4**, 193–200 (1994).

26. Davis, A. L., Keeler, J., Laue, E. D. & Moskau, D. Experiments for recording pure-absorption heteronuclear correlation spectra using pulsed field gradients. *J. Magn. Reson.* **98**, 207–216 (1992).

27. Kay, L. E., Xu, G. Y., Singer, A. U., Muhandiram, D. R. & Forman-Kay, J. D. A Gradient-Enhanced HCCH-TOCSY Experiment for Recording Side-Chain 1H and 13C Correlations in H2O Samples of Proteins. *J. Magn. Reson.* **101**, 333–337 (1993).

28. Diercks, T., Coles, M. & Kessler, H. An efficient strategy for assignment of cross-peaks in 3D heteronuclear NOESY experiments. *J. Biomol. NMR* **15**, 177–180 (1999).

29. Schleucher, J. *et al.* A general enhancement scheme in heteronuclear multidimensional NMR employing pulsed field gradients. *J. Biomol. NMR* **4**, 301–306 (1994).

30. Kay, L. E., Keifer, P. & Saarinen, T. Pure Absorption Gradient Enhanced Heteronuclear Single Quantum Correlation Spectroscopy with Improved Sensitivity. *J. Am. Chem. Soc.* **114**, 10663–10665 (1992).

31. Kay, L. E., Torchia, D. A. & Bax, A. Backbone Dynamics of Proteins As Studied by 15N Inverse Detected Heteronuclear NMR Spectroscopy: Application to Staphylococcal Nuclease. *Biochemistry* **28**, 8972–8979 (1989).
