# Supplementary material for: Characterization of a fold in TANGO1 evolved from SH3 domains for the export of bulky cargos
Source: Nat Commun. 2023 Apr 20;14:2273. doi: 10.1038/s41467-023-37705-4 (PMC10119292; doi:10.1038/s41467-023-37705-4)
Supplement: Supplementary file 2 — Reporting Summary [file 41467_2023_37705_MOESM2_ESM.pdf]

nature portfolio

Corresponding author(s): Raphael Stoll

Last updated by author(s): Mar 8, 2023

Reporting Summary

Nature Portfolio wishes to improve the reproducibility of the work that we publish. This form provides structure for consistency and transparency in reporting. For further information on Nature Portfolio policies, see our Editorial Policies and the Editorial Policy Checklist.

Statistics

For all statistical analyses, confirm that the following items are present in the figure legend, table legend, main text, or Methods section.

n/a	Confirmed	
☐	☒	The exact sample size (*n*) for each experimental group/condition, given as a discrete number and unit of measurement
☐	☒	A statement on whether measurements were taken from distinct samples or whether the same sample was measured repeatedly
☒	☐	The statistical test(s) used AND whether they are one- or two-sided<br>*Only common tests should be described solely by name; describe more complex techniques in the Methods section.*
☒	☐	A description of all covariates tested
☒	☐	A description of any assumptions or corrections, such as tests of normality and adjustment for multiple comparisons
☐	☒	A full description of the statistical parameters including central tendency (e.g. means) or other basic estimates (e.g. regression coefficient) AND variation (e.g. standard deviation) or associated estimates of uncertainty (e.g. confidence intervals)
☒	☐	For null hypothesis testing, the test statistic (e.g. *F*, *t*, *r*) with confidence intervals, effect sizes, degrees of freedom and *P* value noted<br>*Give P values as exact values whenever suitable.*
☒	☐	For Bayesian analysis, information on the choice of priors and Markov chain Monte Carlo settings
☒	☐	For hierarchical and complex designs, identification of the appropriate level for tests and full reporting of outcomes
☒	☐	Estimates of effect sizes (e.g. Cohen's *d*, Pearson's *r*), indicating how they were calculated

Our web collection on statistics for biologists contains articles on many of the points above.

Software and code

Policy information about availability of computer code

Data collection	TopSpin 3.6.1 and 4.1.4, MO.Control software version 2.3 (Nano Temper Technologies)
Data analysis	CcpNMR Analysis 2.4.2, ARIA 2.3.1, nmrPipe 9.8, TITAN 1.6, TALOS+, Clustal Omega (EBI tools webservice), MO.Affinity software version 2.3 (Nano Temper Technologies)

For manuscripts utilizing custom algorithms or software that are central to the research but not yet described in published literature, software must be made available to editors and reviewers. We strongly encourage code deposition in a community repository (e.g. GitHub). See the Nature Portfolio guidelines for submitting code & software for further information.

Data

Policy information about availability of data

All manuscripts must include a data availability statement. This statement should provide the following information, where applicable:

- Accession codes, unique identifiers, or web links for publicly available datasets
- A description of any restrictions on data availability
- For clinical datasets or third party data, please ensure that the statement adheres to our policy

Protein sequences with the following accession codes were downloaded from the UniProt and NCBI database (Q5JRA6, Q0VC16, Q8BI84, F1R5N2, Q9VMA7, A0A5B7CJZ6, A0A5N5SML6, Q96PC5, A0A3Q1LM15, Q91ZV0, A5PLB3, Q9NRC9, Q16674, P12931, P62993, P00519, P06241, LOC412103, P08572, P29400, P53420, Q01955, Q14031). The NMR assignments for TANGO1(30-139) from Drosophila melanogaster and human MOTH domain of TANGO1(21-131) are deposited in the

Biological Magnetic Resonance Bank (https://bmrb.io/) under accession codes 51871 [https://bmrb.io/data_library/summary/index.php?bmrbId=51871] and 34708 [https://bmrb.io/data_library/summary/index.php?bmrbId=34708]. The atomic coordinates for the structure in solution of human TANGO1(21-131) have been deposited with the Protein Data Bank under accession code 7R3M [https://www.rcsb.org/structure/7R3M].

Human research participants

Policy information about studies involving human research participants and Sex and Gender in Research.

Reporting on sex and gender	not applicable
Population characteristics	not applicable
Recruitment	not applicable
Ethics oversight	not applicable

Note that full information on the approval of the study protocol must also be provided in the manuscript.

Field-specific reporting

Please select the one below that is the best fit for your research. If you are not sure, read the appropriate sections before making your selection.

☒ Life sciences ☐ Behavioural & social sciences ☐ Ecological, evolutionary & environmental sciences

For a reference copy of the document with all sections, see nature.com/documents/nr-reporting-summary-flat.pdf

Life sciences study design

All studies must disclose on these points even when the disclosure is negative.

Sample size	As per convention, the NMR backbone 15N {1H}-heteronuclear NOE I/I0 data +/- SD are derived from n=1 biological sample measured in triplicate.
Data exclusions	In NMR, dihedral restraint violations between 5 ° and 12 ° were excluded from the calculation, because the uncertainty of the phi- and psi-torsion angles predicted by TALOS+ was reported as 12.6 ° and 12.3 °, respectively. In MST, capillaries displaying aggregation or adsorption were excluded according to the manufacturer's instructions.
Replication	Biochemical preparations were repeated three to five times with similar results. NMR backbone 15N {1H}-heteronuclear NOE I/I0 data were derived from n=3 independent experiments.
Randomization	Randomization was not applicable as this study does not deal with a cohort.
Blinding	Blinding was not applicable as this study does not deal with a cohort.

Reporting for specific materials, systems and methods

We require information from authors about some types of materials, experimental systems and methods used in many studies. Here, indicate whether each material, system or method listed is relevant to your study. If you are not sure if a list item applies to your research, read the appropriate section before selecting a response.

Materials & experimental systems

n/a	Involved in the study	
☒	☐	Antibodies
☒	☐	Eukaryotic cell lines
☒	☐	Palaeontology and archaeology
☒	☐	Animals and other organisms
☒	☐	Clinical data
☒	☐	Dual use research of concern

Methods

n/a	Involved in the study	
☒	☐	ChIP-seq
☒	☐	Flow cytometry
☒	☐	MRI-based neuroimaging
